# The Diagnostic Usefulness of Circulating Profile of Extracellular Matrix Components: Sulfated Glycosaminoglycans (sGAG), Hyaluronan (HA) and Extracellular Part of Syndecan-1 (sCD138) in Patients with Crohn’s Disease and Ulcerative Colitis

**DOI:** 10.3390/jcm10081722

**Published:** 2021-04-16

**Authors:** Alicja Derkacz, Paweł Olczyk, Agnieszka Jura-Półtorak, Krystyna Olczyk, Katarzyna Komosinska-Vassev

**Affiliations:** 1 Department of Clinical Chemistry and Laboratory Diagnostics, Faculty of Pharmaceutical Sciences in Sosnowiec, Medical University of Silesia in Katowice, 41-200 Sosnowiec, Poland; derkacz.alicja@gmail.com (A.D.); ajura@sum.edu.pl (A.J.-P.); olczyk@sum.edu.pl (K.O.); 2 Department of Community Pharmacy, Faculty of Pharmaceutical Sciences in Sosnowiec, Medical University of Silesia in Katowice, 41-200 Sosnowiec, Poland; polczyk@sum.edu.pl

**Keywords:** inflammatory bowel diseases (IBD), Crohn’s disease (CD), ulcerative colitis (UC), sulfated glycosaminoglycans (sGAG), hyaluronan (HA), soluble part of syndecan-1 (sCD138)

## Abstract

The described research focused on the diagnostic usefulness of sulfated glycosaminoglycans (sGAG), hyaluronan (HA), and extracellular part of syndecan-1 (sCD138) as new markers related to extracellular matrix (ECM) remodeling in the intestine during the two most common forms of inflammatory bowel diseases (IBD), i.e., ulcerative colitis (UC) and Crohn’ disease (CD). Inflammatory markers belonging to ECM components were assessed in serum of patients with IBD using an immunoenzymatic method (HA and sCD138) and a method based on the reaction with dimethylmethylene blue (sulfated GAG). Measurements were carried out twice: at baseline and after one year of therapy with prednisone (patients with CD) or adalimumab (patients with UC). No quantitative changes were observed in serum sGAG, HA, and sCD138 concentrations between patients newly diagnosed with CD and the healthy group. In the case of patients with UC, the parameter which significantly differentiated healthy subjects and patients with IBD before biological therapy was HA. Significant correlation between serum HA level and inflammation activity, expressed as Mayo score, was also observed in patients with UC. Moreover, the obtained results have confirmed that steroid therapy with prednisone significantly influenced the circulating profile of all examined ECM components (sGAG, HA, and sCD138), whereas adalimumab therapy in patients with UC led to a significant change in only circulating sGAG levels. Moreover, the significant differences in serum HA levels between patients with UC and CD indicate that quantification of circulating HA may be useful in the differential diagnosis of CD and UC.

## 1. Introduction

In the last decades, global, dynamic growth in the frequency of chronic inflammatory bowel diseases (IBD) has been observed, further highlighting the role of environmental factors in this disease. Despite significant advances in treatment and its increased availability, the diseases still remain a challenge for modern medicine [1]. Among the inflammatory bowel diseases, the two most significant ones from a clinical standpoint are Crohn’s disease (CD) and ulcerative colitis (UC). This group of diseases was most likely already recognized in ancient times, which is implied by the preserved descriptions. Thus, it can be assumed that their basis is not determined solely by the characteristics of modern lifestyle and environmental factors [2]. IBD etiology and pathogenesis is not yet completely understood, which leads to a lack of accurate methods for causal treatment. Difficulties in identifying the factors responsible for provoking and aggravating inflammatory bowel diseases are the result of, among others, their varied genetical basis, multiple environmental factors, including stress and bacterial and viral infections, which often overlap on clinical images [3]. Furthermore, diagnosis IBD is a significant challenge, and the diagnostic process requires a joint analysis of data from medical history, physical examination, and laboratory tests. A key role in diagnosis is played by endoscopic examination along with mucous membrane biopsy followed by a histologic examination of the obtained sample [4,5]. In clinical practice, there is still a lack of non-invasive, reliable, and objective methods supporting diagnostic methods that allow differentiation, assessment of disease activity, and monitoring of treatment and prognosis of the disease course. Among them, biochemical markers related with the extracellular matrix (ECM) represent promising candidates that can support CD and UC diagnostics. Quantitative changes in the composition and structure of ECM components has been described as playing a significant role in the pathogenesis of inflammatory bowel diseases. Thus, determining the concentration of these macromolecules in body fluids, i.e., serum or urine, is finding increasingly more applications during clinical diagnostics, often being a marker of disease activity and correlating with the severity of the disease process. Hence, establishing the relationship between the level of the circulating matrix component and the progression of IBD might be an argument for the implementation of its determination in the assessment of the activity and progression of IBD [6,7,8,9,10].

Among the new markers related to the ECM remodeling in the intestine proteoglycans (PGs) and their sugar components, glycosaminoglycans (GAGs) are of particular interest due to their role in IBD progression. Sulfated GAGs, including chondroitin/dermatan sulfates, heparan sulfates, and keratan sulfates, integrate the structure of the extracellular matrix by interacting with its components [11]. They are also a reservoir for cytokines and growth factors, take part in regulating the progression of inflammatory processes, accelerate wound healing, and take part in morphogenesis [12,13]. The anticoagulative properties of heparan sulfate and heparin are a result of their ability to bind platelet factors and activate lipoprotein lipase. Due to these properties, GAGs play a significant role in the progression of immunological and inflammatory processes, including intestinal inflammation during IBD [10,13]. Apart from GAGs, the process of extracellular matrix remodeling throughout IBD also involves hyaluronan (HA), a non-sulfated glycosaminoglycan which actively participates in wound healing, proliferation, and migration of cells and modulation of the inflammatory process. Due to its ability to bind water within a polysaccharide net, it provides adequate hydration to the matrix, which allows, e.g., cell migration and substance diffusion [14]. During inflammatory conditions such as IBD, HA polymers are cleaved into fragments of low molecular weight that induce signaling of inflammatory responses via specific receptors. Moreover, during IBD, HA also forms complexes with trypsin inter-α-inhibitor, which are highly adhesive towards leukocytes and additionally exacerbate the inflammation [15].

Another significant component of the extracellular matrix, which influences the progression of inflammation processes including those associated with IBD, is syndecans–PGs belonging to the family of type 1 transmembrane heparan sulfate proteoglycans. The biological functions of syndecans, related to their structure, include cell adhesion, activation and binding of growth factors, and interaction with many ligands, including fibroblast growth factor, vascular endothelium growth factor, fibronectin, and antitrombin-1 [16,17]. Four syndecans have been recognized on the basis of their structure and origin: 1, 2, 3, and 4 [17]. The most interesting one in the context of IBD pathogenesis is syndecan-1 (CD138). It is expressed at high rates in vascular endothelium and circulatory cells. It can also be found in immature B cells, where it is regulated by IL-6 and bacterial lipopolysaccharides (LPS). Syndecan-1, as a co-receptor of multiple extracellular ligands including proinflammatory cytokines and growth factors, plays a significant role in inflammatory processes [17,18]. An extracellular part of syndecan-1 (sCD138) is proteolytically released from the cell surface. This process is activated in response to inflammation and pathogen infection [17]. It is therefore clearly implied that the presence of the extracellular domain of syndecan-1 in blood could be a precious marker of the activity of inflammatory processes [19,20,21]. For this reason, the aim of this article was to evaluate the diagnostic suitability of the serum profile of extracellular matrix components such as sulfated glycosaminoglycans (sGAG), hyaluronan (HA), and soluble syndecan-1 (sCD138) fragments in patients with inflammatory bowel diseases, such as Crohn’s disease and ulcerative colitis.

## 2. Materials and Methods

### 2.1. Study Population

The investigated biological material consisted of venous blood samples obtained from 87 subjects including 40 healthy individuals and 47 patients with inflammatory bowel diseases, such as Crohn’s disease and ulcerative colitis. The diagnosis was made on the basis of clinical symptoms, laboratory test and, in particular, the results of colonoscopy. Additionally, in the case of UC patients, assessment with the Mayo score was used.

### 2.2. Patients with Ulcerative Colitis (UC)–Inclusion and Exclusion Criteria

UC patients enrolled in this study included a group of 31 patients selected for a clinical research program involving biological treatment with adalimumab (Humira, Abbott GmbH, Wiesbaden, Federal Republic of Germany). Adalimumab bonds specifically with tumor necrosis factor (TNF), restricting its activity by blocking it from binding with p55 and p75 TNF receptors on the cell surface. This antibody also influences the biological response to TNF and impacts the concentrations of intercellular adhesion molecules responsible for leukocyte migration (ELAM-1, VCAM-1, and ICAM-1). In the case of patients with UC, the inclusion criteria included age between 18 and 75 during the control visit, diagnosed ulcerative colitis, diagnosed active form of ulcerative colitis confirmed through colonoscopy with biopsy, active form of ulcerative colitis confirmed through Patient Global Assessment (PGA) 2 or 3, and treatment with corticosteroids or immunosuppressive medicine (azathioprine (Imuran, Aspen Pharma Trading Ltd, Dublin, Ireland)) or 6-mercaptopurine (Mercaptopurinum VIS, Zakłady Chemiczno-Farmaceutyczne „VIS”® Spółka z o.o., Bytom, Poland)) which had not brought the intended results according to the practitioner’s opinion.

The criteria for exclusion, in the case of patients with UC, were colectomy with ileorectostomy or colectomy with anastomosis, Kock pouch or ileostomy undertaken in the course or inflammatory bowel disease or before a planned bowel surgery, adalimumab treatment or former participation in clinical research involving adalimumab, exposure to infliximab or any other TNF inhibitor within 56 days from week 0, taking ciclosporin, tacrolimus, or mycophenolate mofetil within 30 days from week 0, intravenous corticosteroids within 14 days from the screening or throughout the screening, diagnosed fulminant colitis and/or toxic colitis, and taking any biological medicine which could potentially influence the disease under investigation. Adalimumab doses delivered to patients with UC were varied, and the patients were randomly assigned to one of two groups: the first received 160 mg of adalimumab in the initial dose, with a reduction to 80 mg and then to 40 mg, and there was a need for a faster response to treatment in these patients; the second group received 160 mg in the initial dose, with a reduction to 40 mg. The mean time from diagnosis was one year. Moreover, in patients with ulcerative colitis who were using prednisone (Encorton, Pabianickie Zakłady Farmaceutyczne Polfa S.A., Pabianice, Poland) at the time of qualifying for biological treatment after introducing biological drugs, the dose of prednisone was gradually reduced. The cigarette smoking status has not been established in patients with UC enrolled in this study. 

### 2.3. Patients with Crohn’s Disease (CD)—Inclusion and Exclusion Criteria

The next group of patients with IBD in the investigation included 16 people with CD, treated mainly with prednisone. The dosage of prednisolone was chosen on a case-by-case basis, depending on the response to treatment. Blood samples were obtained from patients at the point of diagnosis and again after a year of treatment. The following inclusion criteria have been adapted for the volunteers: age above 18 and diagnosed with Crohn’s disease (CD). The exclusion criteria were age under 18, diagnosed indeterminate colitis (IC), alcoholism, alcohol-related liver disease or any ongoing liver disease, acute infections of viral, fungal, or bacterial origin, mild or acute myocardial inefficiency, unstable coronary artery disease, chronic respiratory failure, chronic kidney disease, chronic liver disease, demyelinating disease, diabetes, pregnancy or breastfeeding, and diagnosed precancerous condition or cancerous comorbidities. The patients had been ill for twelve months with a period of activity and remission and did not take any additional medications during therapy. The cigarette smoking status has not been established in patients with CD enrolled in this study. None of the patients had a prior ileocolonic resection for CD and perianal fistulizing CD.

All patients were treated at the Department of Gastroenterology of St. Barbara’s Regional Specialist Hospital in Sosnowiec. Reference number of the decision by the Bioethics Committee at the Medical University of Silesia in Katowice: KNW/0022/KB/309/15.

### 2.4. Control Subjects

The reference material for research consisted of blood samples obtained from 40 healthy people in properly sampled age groups. The group qualified for the research involved people who had not been hospitalized during the prior year, had not undergone surgical treatment, and had not been pharmacologically treated. Furthermore, the results of their routine laboratory tests (i.e., complete blood count, erythrocyte sedimentation rate test (ESR test), plasma fasting glucose, fasting lipid profile, creatinine, liver enzymes, rheumatoid factor (RF), and CRP) were within the reference values for their particular age group. Subjects were excluded from the study if they had been taking steroidal or non-steroidal anti-inflammatory drugs. None of the volunteers were cigarette smokers or had any history of drug or alcohol abuse.

### 2.5. Biological Material for Research

Blood sampled from the elbow vein, contained in test tubes with no added anticoagulant, was centrifuged for 10 min at 1500× *g* at 4 °C. The serum obtained through centrifugation was subjected to diagnostic examination as requested by the attending physician. The remaining part was frozen and stored at −80 °C until needed for biochemical analysis.

### 2.6. Assessing the Serum Hyaluronan (HA) Concentration

Hyaluronan concentration was determined using a using enzyme-linked immunosorbent assays test supplied by TECOmedicalGroup TE 1018-2, Sissach, Switzerland. The analytical sensitivity of the method for HA concentrations assessing was 2.7 ng/mL and the intra-run error was 6.4%.

### 2.7. Assessing the Serum Soluble Part of Syndecan-1 (sCD138) Concentration

The concentration of the extracellular part of syndecan-1 circulating in blood was evaluated with an immunoassay test supplied by Diaclone SAS, BesanconCedex, France. The analytical sensitivity of the method for sCD138 concentrations assessing was 4.94 ng/mL and the intra-run error was 6.2%.

### 2.8. Assessing the Serum Sulfated Glycosaminoglycans (sGAG) Concentration 

Sulfated glycosaminoglycans were assessed using the Wieslab^®^ sGAG quantitative test (GAG 201 RUO, Euro Diagnostica AB, Malmo, Sweden). The principle of the method is based on specific interaction between sulfated glycosaminoglycans (chondroitin sulfates, dermatan sulfates, keratan sulfates, heparan sulfates, and heparin) and the tetravalent cationic Alcian blue. The reagents for this kit, i.e. Alcian Blue stock solution (containing 0.1% H_2_SO_4_ and 0.4 M GuHCl), 8 M Guanidine-HCl, SAT solution (containing 0.3% H_2_SO_4_ and 0.75 Triton X-100), DMSO solution (containing 40% dimethylsulphoxide and 0.05 M Mg_2_Cl_2_), Gu-Prop solution containing 4 M GuHCl, 33% 1-propanol and 0.25% Triton X-100, calibrators containing 1.1 mL chondroitin sulphate-6 were manufactured by Euro Diagnostica AB from Malmo, Sweden. The assay was performed at pH low enough to neutralize all carboxylic and phosphoric acid groups as well as at ionic strength large enough to eliminate ionic interactions other than those between Alcian blue and sulfated GAGs. Six calibrators containing chondroitin-6-sulfate at concentrations of 12.5, 25, 50, 100, 200, and 400 μg/mL were used. Then, 50 µL of the prepared standard solutions for control and test samples were added to the to the appropriate wells on the microplate. Then, 50 µL of 8 M guanidine-HCl solution (GuHCL) was added, the microplate was covered with sealing tape and incubated for 15 min at room temperature. In the next step, 50 µL of the SAT solution containing 0.3% H_2_SO_4_ and 0.75% Triton X-100 was added and incubated for 15 min at room temperature. Then, 750 µL of Alcian blue was added and incubated for 15 min at room temperature. The samples were then centrifuged for 15 min at 12,000× *g*. The next step was to remove the supernatant and add 500 µl of DMSO solution containing 40% dimethylsulfoxide and 0.05 M MgCl_2_ to the pellets. Prepared samples were incubated for 15 min at room temperature, followed by centrifugation for 15 min at 12,000× *g*. After removing the supernatant, 500 µL Gu-Prop solution containing 4 M guanidine-HCl, 33% 1-propanol and 0.25% Triton X-100 was added to the pellets and mixed for 15 min on a shaker. The absorbance of the standard and test samples was measured using a microplate reader at a wavelength of 620 nm.

### 2.9. Statistical Analysis

The statistical analysis of the obtained results was performed using the STATISTICA software, version 10.0, developed by StatSoft, Krakow, Poland. It involved verifying the normality of distribution of the variable using the Shapiro–Wilk test. The descriptive statistics for non-normally distributed variables included the median (Me) as a measure of position and the interquartile range—between the lower (Q1) and the upper (Q3) quartiles—as a typical measure of variability. To verify the hypotheses related to the influence of treatment on the value of the investigated parameters, the pre- and post-treatment results were compared using the Wilcoxon test. The statistical significance of the difference in results between the affected group and the control group was determined using the Mann–Whitney U test. The pre- and post-treatment clinical parameters have been compared using the Wilcoxon test and the paired *t*-test. A significance level of *p* < 0.05 was used for all tests and statistical analyses.

## 3. Results

### 3.1. Research Data

The clinical characteristics of patients with CD and UC before treatment and after a year of treatment has been summarized in Table 1 and Table 2.

It is interesting to note that the patients with CD had no change in standard markers of inflammation following intervention. The results of circulating levels of serum sulfated glycosaminoglycans (sGAG), hyaluronan (HA), and the soluble part of syndecan-1 (sCD138) in study individuals (control subjects, patients with CD, and patients with UC) are summarized and provided as supplementary material (Table S1).

### 3.2. Quantitative Changes of Serum sGAG, HA, and sCD138 in Patients with Crohn’s Disease

The investigation has shown that sGAG and HA concentration in patients newly diagnosed with Crohn’s disease is not significantly different from the concentration of these parameters in the serum of healthy people. A year-long prednisone treatment, on the other hand, caused a significant increase in both sGAG and HA concentrations in the serum of affected patients compared with pre-treatment values. The increase reached 42% for sGAG and 78% for HA. Furthermore, the differences in the concentration of sulfated GAGs and HA between patients with CD after a year of prednisone treatment and the control group were statistically significant. The increase in concentration reached 53% for sGAG and 41% for HA.

The quantitative evaluation of extracellular sydecan-1 (sCD138) component circulating in blood has shown a decrease in the concentration of this ECM component in the blood of patients newly diagnosed with CD who have not undergone treatment compared with the concentration in the blood of healthy people. The difference, however, was not statistically significant (*p* > 0.05). Moreover, a significant, almost 110% increase in circulating sCD138 was observed in patients with CD after a year-long treatment compared with pre-treatment concentrations. 

### 3.3. Quantitative Changes of Serum sGAG, HA, and sCD138 in Patients with Ulcerative Colitis

In the case of patients with UC before implementing biological therapy, the changes to the serum profile of the analyzed ECM components were not statistically different from the concentrations of these components in healthy people, with the exception of HA. Before adalimumab treatment, patients with UC had significantly increased HA concentrations compared with the control group. A year-long treatment with biological medicine, on the other hand, only affected the circulating profile of sulfated GAGs. A statistically significant increase in sGAG concentration was observed in patients with UC compared with the pre-treatment concentration of these ECM components in the same patients. Moreover, patients with ulcerative colitis before therapy had significantly different HA concentration in the blood than patients with CD before steroid therapy implementation.

**Figure 1:**
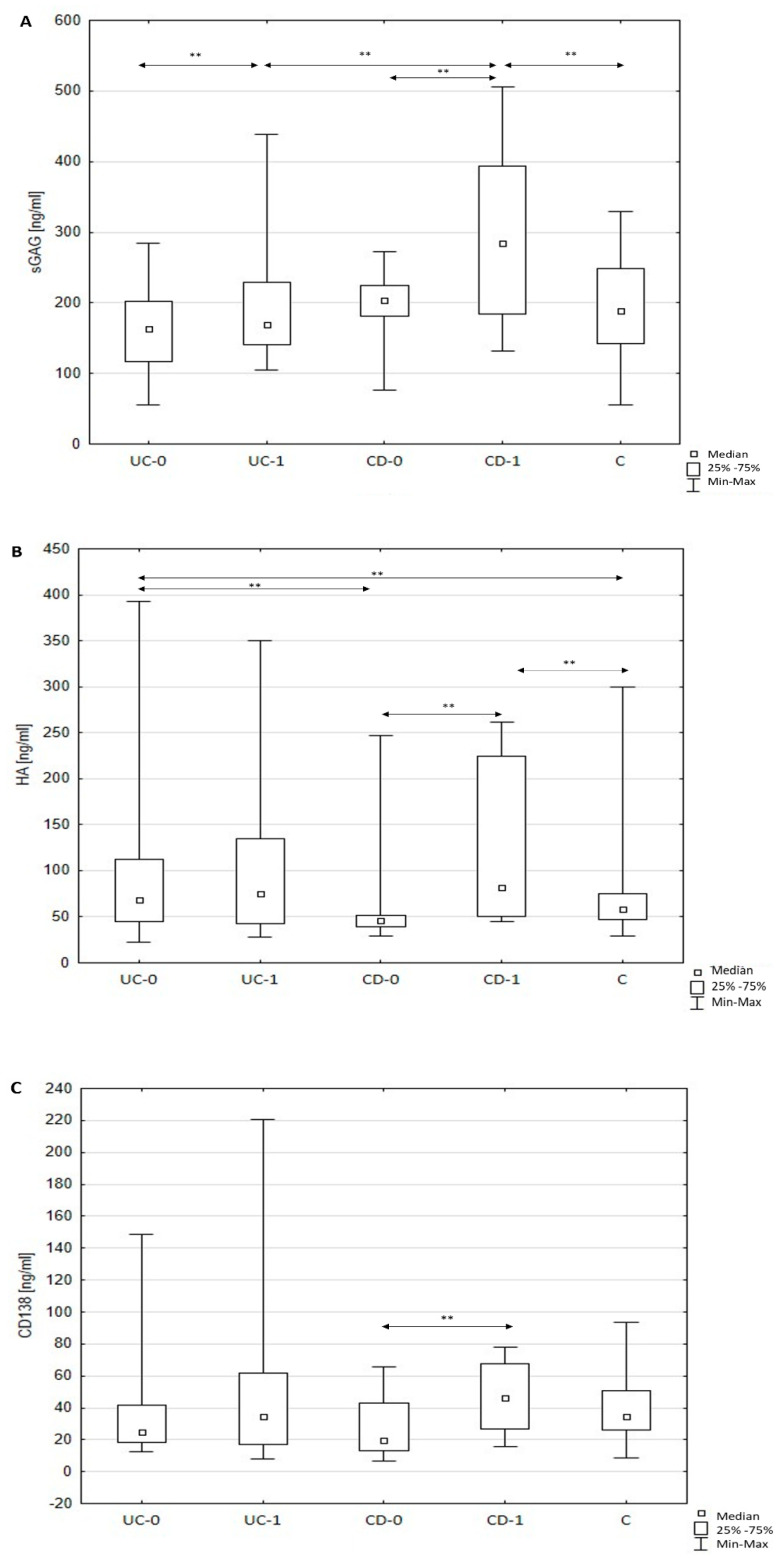
(**A**–**C**) Circulating total sulfated glycosaminoglycans (GAGs), hyaluronic acid (HA), and soluble syndecan-1 (sCD138) levels in healthy subjects and patients with inflammatory bowel diseases (IBD) before and after 12-month therapy. Note: Results are expressed as median, interquartile (25th–75th percentiles) range, and minimum and maximum of data in the groups. C: healthy subjects (control); UC-0: patients with ulcerative colitis before adalimumab therapy; UC-1: patients with ulcerative colitis after 12-month adalimumab therapy; CD-0: patients with Crohn’s disease before prednisone therapy; CD-1: patients with Crohn’s disease after 12-month prednisone therapy. HA, hyaluronic acid; sGAG, sulfated glycosaminoglycans; sCD138, soluble syndecan-1; ** *p* < 0.01, statistically significant.

The results are shown in Figure 1.

### 3.4. The Relationship between Serum ECM Components and Inflammatory Processes and Disease Activity

The investigation has also revealed a relationship between disease activity and the circulating profile of ECM components only in the case of patients with UC. In these patients, a correlation between HA concentration in serum and the activity of inflammation, derived from the Mayo score, was observed both before (r = 0.45; *p* < 0.012), and after 12-month treatment (r = 0.42; *p* < 0.039). A correlation between sCD138 levels in serum and the Mayo score was also observed in patients before biological treatment with adalimumab (r = 0.44; *p* < 0.014). Results of the abovementioned correlation analysis are presented as a correlation plot and included in supplementary material (Figure S1).

The relationship between the concentration of the ECM components and a marker of inflammation process commonly used in clinical practice, the CRP protein, was also investigated. The investigation revealed significantly higher CRP concentrations in patients with CD compared to patients diagnosed with UC. A significant effect of 12-month adalimumab treatment inn lowering CRP concentration in the blood of patients with UC was also observed.

## 4. Discussion

### 4.1. Quantitative Changes of the ECM Components (sGAG, HA, and sCD138) in Serum of Patients Newly Diagnosed with CD or UC

The constant effect of damaging factors during IBD progression leads to changes in the structure and function of the extracellular matrix [22,23]. Our investigation has shown that disturbances affecting the extracellular matrix in the connective tissue during IBD are reflected in quantitative changes of the composition of matrix components in the serum. In the case of patients newly diagnosed with CD, there was no difference in sGAG, HA, and sCD138 concentrations in their serum compared with that of healthy people. In patients with UC, the parameter which was significantly different for healthy people compared with patients before biological medicine treatment was the concentration of hyaluronan in the serum. During physiological states, hyaluronan plays a significant regulatory role in providing ECM integrity, while the depolymerized, small molecule HA fragments generated in IBD play a significant role as inflammatory factors [15]. Signaling through hyaluronan is a mechanism suggesting the regulatory role of ECM integrity failures in IBD pathogenesis. On the one hand, HA fragments promote wound healing; on the other hand, they also promote fibrotic processes, leading to the proliferation of fibroblasts and differentiating myofibroblasts [14,16,17,24]. The increased accumulation of HA fragments in the intestine is related to the inflammation which affects intestinal tissues during IBD progression. It has also been demonstrated that HA takes part in inducing the infiltration of leukocytes into the bowel and activates the immunological response [16,17]. These mechanisms explain the results of the investigation, which suggest increased concentration of HA in the blood of patients with UC before biological treatment. The research so far suggests that increased hyaluronan concentration in the blood and its accumulation in the intestines facilitates IBD progression by increasing the flow of leukocytes into the intestines [17]. Furthermore, HA binds coagulation factors and increases platelet recruitment, which facilitates and supports inflammation during IBD [10]. The research by Kessler et al. [24] points to a significant increase in the concentration of hyaluronan-associated protein (SHAP-HA) in the serum of patients with UC compared to patients with sustained remission, and this value was positively correlated with endoscopic damage. Importantly, the serum SHAP-HA level in patients with CD was correlated only with TNF-α [24]. A difference in HA turnover between UC and CD patients was also observed during our investigation. The significant difference between HA concentration in the serum of patients newly diagnosed with CD and the concentration of this glycan in the serum of patients with UC before treatment suggests the possibility of measuring HA levels during the differential diagnosis of these two very similar types of bowel inflammation, Crohn’s disease and ulcerative colitis. Increased deposition of HA in bowel tissues and its increased transfer into circulation are active participants in the chronic inflammation typical for UC. Quantitative assessment of HA in blood may be an early marker of active disease. Another argument supporting this hypothesis is the fact that our results indicate that HA concentration in the blood of UC patients was correlated with the intensity of the disease as determined from the Mayo score.

### 4.2. The Influence of Therapy with Prednisone (Patients with CD) or Biological Treatment with Adalimumab (Patients with UC) on the Circulating Profile of Analyzed ECM Components

The performed analyses implied that year-long prednisone treatment applied to patients with CD caused an increase in the concentration of all investigated ECM components in the serum (sGAG, HA, and sCD138) compared with pre-treatment values. In the case of patients with UC, year-long treatment with a TNF-α inhibitor also caused a significant increase in sGAG concentration in serum; however, it did not influence the amount of circulating HA and sCD138. The obtained results confirmed that ECM components are an active part of the progression of the IBD disease process, which is possible to regulate by the type of pharmacological treatment. Sulfated GAGs proved to be the matrix components which undergo significant changes in response to the applied treatment both in CD and UC patients. The effect of the observed changes, however, was much more noticeable in patients with CD. Moreover, the investigation revealed that sGAG levels in patients with IBD after a year of proper treatment were significantly higher in patients with CD than in patients with UC, which clearly differentiates the two groups of inflammatory bowel diseases. It has also been shown that steroid-based treatment significantly modulated the level of soluble syndecan-1 (sCD138), significantly increasing its levels in the blood. This is in accordance with the results obtained by other authors [21]. It is believed that releasing the extracellular domain of syndecan-1 from the intestinal epithelial cells may reduce the intensity of inflammatory bowel diseases and the transmigration of neutrophils by inactivating key inflammatory mediators and reducing the expression of proinflammatory cytokines. The protective effect of syndecan-1 extracellular domain circulating in the blood has been confirmed during research on mice lacking sCD138; when subjected to an experimental colitis, they have exhibited significantly increased mortality, impaired mucous membrane regeneration, and extended inflammatory cell recruitment. Furthermore, treating animals with a functional analog of the sCD138 ectodomain significantly affected the symptoms of inflammation [21].

### 4.3. The Influence of Inflammatory Processes and Disease Activity on ECM Components in Patients with IBD

Due to the fact that IBD are diseases involving an active inflammatory process, this article also analyzed the influence of treatment on the grade of inflammation, derived from the concentration of C-reactive protein in blood. The effect of treatment on disease activity, derived from the Mayo score, was also investigated. Even though CRP is not an innate marker of IBD, it may be an indicator of the intensity of the inflammatory process and help in monitoring the state of patients during the stages of remission and aggravation. The research by Mortensen et al. has shown a relationship between CRP and CD activity. The results indicated a significant increase in CRP levels in patients with an active variation of the disease [25]. The research by Chang et al. also revealed a significant correlation between increased CRP concentration and mild or acute clinical form of the disease [26]. In some studies, however, CRP levels were not associated with the disease activity in small intestinal lesions in patients with CD [27,28]. Solem et al. [27], through their analyses, observed that CRP concentration is within the reference range in 75% of patients with CD, in which endoscopic examinations have not confirmed changes to intestinal mucous membrane. In our investigation, pharmacologically treating patients with CD with prednisone also had no influence on changing the clinical activity of the disease, measured using the CDAI score, or on the inflammatory activity, derived from C-reactive protein concentration. On the other hand, significantly higher CRP concentrations have been observed in patients with CD when compared to patients diagnosed with UC; this is consistent with previous studies which showed that patients with CD generally have higher CRP and IL-6 production than patients with UC [29]. However, the mechanisms responsible for this phenomenon have not yet been fully elucidated. One possible mechanism involves the accumulation of mesenteric fat, a major site of IL-6 and TNF-α synthesis, in patients with CD [30]. In our research, the patients with Crohn’s disease had no change in markers of inflammation after one year of treatment. This observation may indicate resistance to glucocorticoid treatment or the presence of fistulas. In the case of patients with ulcerative colitis, CRP concentrations observed during our investigation have therefore been—similarly to the results obtained by other authors—significantly lower than in patients with Crohn’s disease, which suggests a potential role for CRP markers in differentiating these two diseases of similar symptomatology. We have also observed that 12-month adalimumab treatment applied to patients with UC decreased the activity of inflammatory processes within intestinal walls, which was reflected in lower CRP concentration and lower disease activity according to the Mayo score. This result was connected to reduced symptoms of the disease after 12-month treatment using a biological medicine. These observations are consistent with the results by Tursi et al. [31], who have researched the effectiveness and safety of treating patients with UC with adalimumab. They have noticed a similar decrease in CRP concentration and reduced disease activity resulting from the biological treatment [31]. The data suggest that a year-long treatment with a biological medicine results in lower activity of the inflammatory process, which is reflected in the results of endoscopic examination. Furthermore, a correlation was observed between hyaluronan concentration in the blood of patients with UC, both before and after 12-month treatment, and the activity of the disease derived from the Mayo score, which supports the hypothesis that hyaluronan is an active participant in the restructuring of the intestinal mucous membrane, related to chronic bowel inflammation. Hyaluronan, along with the enzymes and binding proteins responsible for its synthesis and degradation, may directly modulate the progression of the disease by controlling the recruitment of immune cells and releasing proinflammatory cytokines [19,24]. These attributes of hyaluronan allow its molecules to take part both in the progression and the remission of the disease. It makes HA molecules and, indirectly, the products of its degradation, perfect candidates for the role of UC progression markers.

## 5. Conclusions

The significant difference in HA concentration in the blood of UC and CD patients suggests the possibility of measuring HA levels during the differential diagnosis of these two very similar types of bowel inflammation. Increased deposition of HA in bowel tissues and its increased transfer into circulation are active participants in the chronic inflammation typical for UC. Quantitative changes of HA in blood may be an early marker of active disease. Moreover, our study confirmed that ECM components are an active part of the progression of the IBD disease process, which is possible to regulate according to the type of pharmacological treatment.

The results obtained during this investigation reveal a variety of mechanisms regulating the activity of the disease and the restructuring of extracellular matrix components as well as the potential usefulness of circulating ECM components as markers for evaluating ECM remodeling during IBD and for monitoring pharmacological treatment. Finding potentially useful diagnostic markers of changes occurring throughout IBD, determined in readily available biological material, may provide an aid to the clinical diagnosis and/or monitoring the progression of treatment. However, further research should be performed on a larger group of patients in order to confirm the obtained results. Further investigation could possibly lead to identifying a component of extracellular matrix that will meet the criteria of an ideal marker and will protect patients from invasive colonoscopy examination.

## Figures and Tables

**Table 1:** Clinical characteristics of patients with ulcerative colitis (UC).

Parameter	Patients with Ulcerative Colitis		*p*
	before Treatment UC_0_	after Treatment UC_1_	UC_0_ Vs. UC_1_
Age (years)	33.38 ± 12.75		
Mayo score	3 (2–3)	2 (1–3)	0.000
CRP (mg/L)	3.37 (0.79–26.44)	2.41 (1.42–7.33)	0.031
Glucose (mmol/L)	4.99 ± 0.72	4.81 ± 0.81	0.331
Cholesterol (mmol/L)	4.91 ± 0.89	4.99 ± 0.86	0.264
Triglycerides (mmol/L)	1.41 ± 0.52	1.14 ± 0.38	0.030
Indirect bilirubin (μmol/L)	5.20 (4.61–8.32)	8.30 (5.50–16.70)	0.000
Direct bilirubin (μmol/L)	3.70 (2.90–4.42)	5.30 (3.51–8.21)	0.010
ALT (U/l)	15.02 (10.04–26.00)	16.01 (10.01–25.03)	0.814
AST (U/L)	19.00 (14.02–21.02)	19.03 (15.02–23.01)	0.100
Total protein (g/L)	73.48 ± 5.43	74.73 ± 5.63	0.235
Albumin (g/L)	42.00 (40.01–46.03)	43.02 (40.03–48.00)	0.264
WBC	7.90 (4.6–13.6)	7.90 (3.9–13.7)	0.61
PLT (× 10^9^/L)	372.03 (292.00–457.00)	343.04 (263.02–422.03)	0.050
BMI	24.25 ± 3.59	24.46 ± 4.23	0.455

Comparison of selected parameters before and after treatment. Data are presented as median, interquartile (25th–75th percentile) range or mean ± standard deviation (SD), or percentage (%). Data analyzed using the Wilcoxon test or paired *t*-test. *p* < 0.05, statistically significant. ALT, alanine aminotransferase; AST, aspartate aminotransferase; BMI, body mass index; CRP, C-reactive protein; PLT, platelets; WBC, white blood cells; UC, ulcerative colitis; UC_0_, patients with ulcerative colitis before adalimumab therapy; UC_1_, patients with ulcerative colitis after 1 year adalimumab therapy.

**Table 2:** Clinical characteristics of patients with Crohn’s disease (CD).

Parameter	Patients with Crohn’s Disease		*p*
	before Treatment CD_0_	after Treatment CD_1_	CD_0_ Vs. CD_1_
Age (years)	32.10 ± 9.56		
CDAI	299.60 ± 47.93	274.01 ± 50.71	0.323
CRP (mg/L)	15.70 (4.22–39.05)	15.20 (5.30–28.90)	0.665
Glucose (mmol/L)	4.89 (4.72–5.47)	4.95 (4.61–5.17)	0.950
Indirect bilirubin (μmol/L)	5.75 (4.85–7.43)	6.25 (5.2–10.65)	0.073
Direct bilirubin (μmol/L)	3.75 (2.88–4.05)	7.9 (4.5–10.8)	0.020
ALT (U/L)	24 (16.25–29.05)	21.5 (14.5–31)	0.351
AST (U/L)	21.5 (18.5–24.25)	21 (16.75–23.50)	0.373
Total protein (g/L)	72.13 ± 4.90	77.25 ± 5.21	0.004
Albumin (g/L)	43.50 (42–47.25)	43.5 (42–49)	0.531
WBC (10^3^/µL)	7.12 ± 3.20	6.68 ± 2.05	0.421
PLT (x10^9^/L)	356.50 (277.50–396.02)	232.20 (134.21–309.11)	0.090
BMI	20.58 ± 3.43	19.84 ± 2.84	0.190

Comparison of selected parameters before and after treatment. Data are presented as median, interquartile (25th–75th percentile) range or mean ± standard deviation (SD), or percentage (%). Data analyzed using the Wilcoxon test or paired *t*-test. *p* < 0.05, statistically significant. ALT, alanine aminotransferase; AST, aspartate aminotransferase; BMI, body mass index; CD, Crohn’s disease, CD_0_, patients with Crohn’s disease before prednisone therapy; CD_1_, patients with Crohn’s disease after 1 year prednisone therapy; CDAI, The Crohn’s disease activity index; CRP, C-reactive protein; PLT, platelets; WBC, white blood cells.

## Data Availability

Data is contained within the article or supplementary material.

## Supplementary Materials

The following are available online at https://www.mdpi.com/article/10.3390/jcm10081722/s1, Figure S1: Results of correlation analysis between disease activity expressed as the Mayo score and serum concentration of the hyaluronan (HA) or soluble syndecan-1 (sCD138) level in patients with ulcerative colitis both before, and after 12-month adalimumab therapy; Table S1: Serum concentrations of sulfated glycosaminoglycans (sGAG), hyaluronic acid (HA) and soluble syndecan-1 (sCD138) in patients with Crohn disease (CD) and ulcerative colitis (UC) at baseline and after one year of therapy.
