# Supplementary material for: The Diagnostic Usefulness of Circulating Profile of Extracellular Matrix Components: Sulfated Glycosaminoglycans (sGAG), Hyaluronan (HA) and Extracellular Part of Syndecan-1 (sCD138) in Patients with Crohn’s Disease and Ulcerative Colitis
Source: J Clin Med. 2021 Apr 16;10(8):1722. doi: 10.3390/jcm10081722 (PMC8073401; doi:10.3390/jcm10081722)
Supplement: Supplementary file 1 [file jcm-10-01722-s001.zip › jcm-1173742-supplementary.pdf]

Table S1. Serum concentrations of sulfated glycosaminoglycans (sGAG), hyaluronic acid (HA) and soluble syndecan-1 (sCD138) in patients with Crohn disease (CD) and ulcerative colitis (UC) at baseline and after one year of therapy

Parameter	Studied groups			p				
	**Control subjects (C)**	**Crohn disease at baseline (CD_0)**	**Crohn disease after 1 year of therapy (CD_1)**	**CD_0 vs C**	**CD_1 vs C**	**CD_0 vs CD_1**	**CD_0 vs UC_0**	**CD_1 vs UC_1**
**sGAG** (ng/mL)	188.80 (142.58 – 249.53)	203.91 (179.38 – 227.17)	289.53 (189.35 – 442.75)	NS	0,013	0,015	NS	0,015
**HA** (ng/mL)	58.02 (47.00 – 74.72)	45.98 (39.10 – 51.92)	81.96 (50.41 – 224.28)	NS	0.038	0,041	0,016	NS
**sCD138** (ng/mL)	34.65 (26.35 – 51.19)	22.03 (16.57 – 42.80)	46.19 (24.33 – 54.97)	NS	NS	0,0005	NS	NS
		**Ulcerative colitis at baseline (UC_0)**	**Ulcerative colitis after 1 year of therapy (UC_1)**	**UC_0 vs C**	**UC_1 vs C**	**UC_0 vs UC_1**		
**sGAG** (ng/mL)	188.80 (142.58 – 249.53)	162.66 (116.57 – 202.35)	168.63 (140.27 –229.04)	NS	NS	0,039		
**HA** (ng/mL)	58.02 (47.00 – 74.72)	68.20 (44.84 – 112.14)	75.42 (42.69 – 134.72)	0.041	NS	NS		
**sCD138** (ng/mL)	34.65 (26.35 – 51.19)	28.14 (19.82 – 47.03)	27.50 (10.98 – 20.96)	NS	NS	NS		

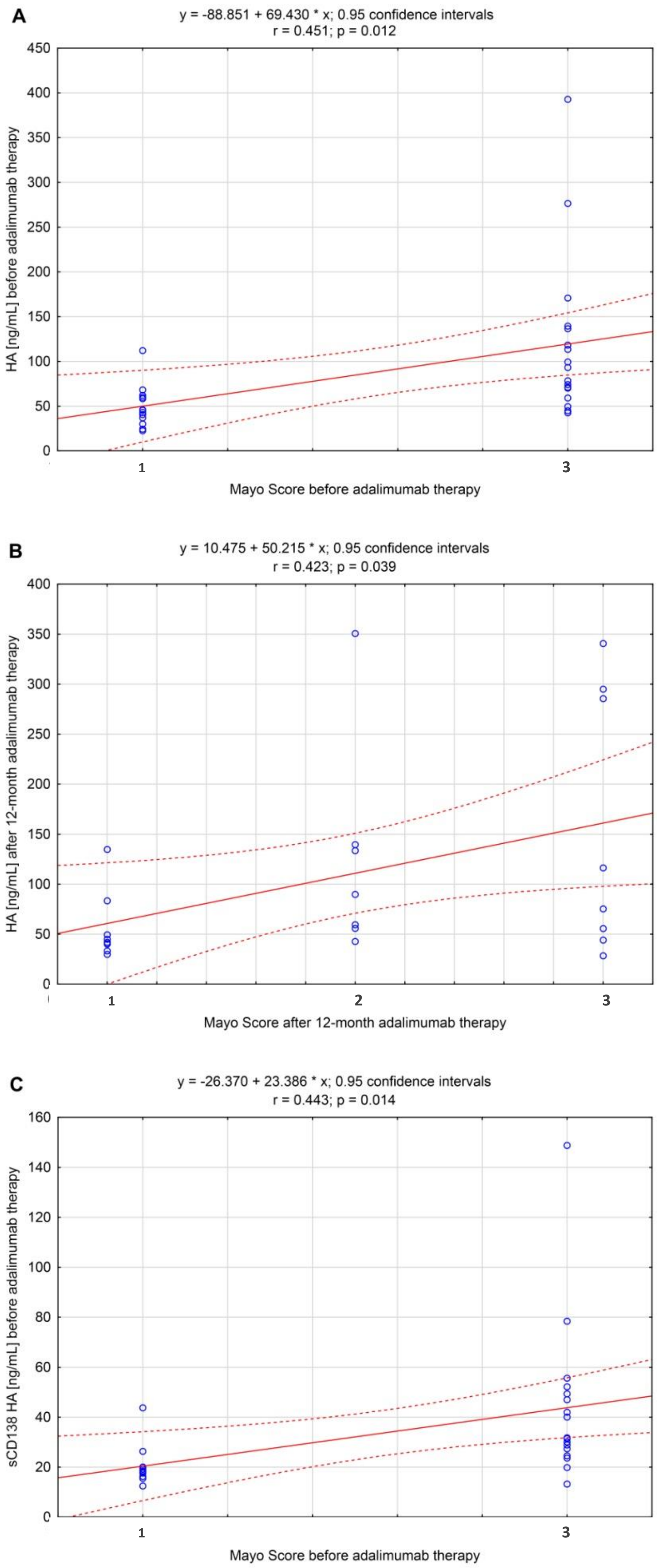

Figure S1. (A-C) Results of correlation analysis between disease activity expressed as the Mayo score and serum concentration of the hyaluronan (HA) or soluble syndecan-1 (sCD138) level in patients with ulcerative colitis both before, and after 12-month adalimumab therapy

Note: HA, hyaluronan; sCD138, soluble syndecan-1; $p < 0.05$, a statistically significant.
